# Does Observation of Postural Imbalance Induce a Postural Reaction?

**DOI:** 10.1371/journal.pone.0017799

**Published:** 2011-03-15

**Authors:** Banty Tia, Arnaud Saimpont, Christos Paizis, France Mourey, Luciano Fadiga, Thierry Pozzo

**Affiliations:** 1 INSERM U887, Motricité et Plasticité, Université de Bourgogne, Dijon, France; 2 Gérontopôle, Centre Hospitalier Universitaire de Dijon, Dijon, France; 3 Centre d'Expertise de la Performance « Gilles Cometti », UFR STAPS, Université de Bourgogne, Dijon, France; 4 Section of Human Physiology, Department SBTA, Faculty of Medicine, University of Ferrara, Ferrara, Italy; 5 RBCS (Robotics, Brain and Cognitive Sciences) Department, Italian Institute of Technology, Genoa, Italy; 6 Institut Universitaire de France, UFR STAPS, Université de Bourgogne, Dijon, France; The University of Western Ontario, Canada

## Abstract

**Background:**

Several studies bring evidence that action observation elicits contagious responses during social interactions. However automatic imitative tendencies are generally inhibited and it remains unclear in which conditions mere action observation triggers motor behaviours. In this study, we addressed the question of contagious postural responses when observing human imbalance.

**Methodology/Principal Findings:**

We recorded participants' body sway while they observed a fixation cross (control condition), an upright point-light display of a gymnast balancing on a rope, and the same point-light display presented upside down. Our results showed that, when the upright stimulus was displayed prior to the inverted one, centre of pressure area and antero-posterior path length were significantly greater in the upright condition compared to the control and upside down conditions.

**Conclusions/Significance:**

These results demonstrate a contagious postural reaction suggesting a partial inefficiency of inhibitory processes. Further, kinematic information was sufficient to trigger this reaction. The difference recorded between the upright and upside down conditions indicates that the contagion effect was dependent on the integration of gravity constraints by body kinematics. Interestingly, the postural response was sensitive to habituation, and seemed to disappear when the observer was previously shown an inverted display. The motor contagion recorded here is consistent with previous work showing vegetative output during observation of an effortful movement and could indicate that lower level control facilitates contagion effects.

## Introduction

There is an increasing amount of research on human sensitivity to the gestural signals made by others and related understanding of others' emotions/intentions [1]. Indeed a number of studies demonstrated motor contagion through facilitation and interference effects of action observation during action execution [2], [3]. Properties of the observed movement such as tempo [4] and speed [5] were also shown to contaminate the ongoing or subsequent movement performed by the observer [6]. Further, the observation of other individuals during social interaction is sometimes sufficient to elicit spontaneous imitation, leading to the well known “chameleon effect” [7], [8]. Such spontaneous imitation was suggested to play a role in action recognition and understanding [8], [9], and to foster mutual liking and empathy [8], [10].

However among all these studies, none tested such contamination when the subject is required to stay standing and immobile. Moreover, even though automatic imitation has been observed in a social context, it is still unclear in which conditions action observation triggers motor behaviours. Despite a spontaneous tendency for imitation, most observed actions are not imitated, suggesting that inhibitory mechanisms prevent motor outputs [11], [12]. For instance, no significant muscle activity is elicited in observers being showed an effortful movement [13]. Such evidence for inhibitory processes is not surprising given that compulsive imitation is extremely costly in energy consumption [11]. Further, compulsive imitative behaviour observed in patients with prefrontal lesions for whom inhibitory pathways are affected [14], [15] leads to severe deficiency in social interactions.

Inhibition of imitative tendencies is particularly relevant to the example of postural imbalance. In this condition, imitation would impair motor efficiency and entail a postural threat for the observer. This raises the question of the sensitivity of imitation in the particular case where inhibition mechanisms in addition to postural control (as a movement restrictor) would penalise imitation. Indeed, upright posture stabilisation mechanisms tend to align the vertical weight force with the resultant ground reaction force, thus reducing the horizontal distance between the centre of mass (CoM, point where the vertical weight force may be considered to apply), and the centre of pressure (CoP, point location of the resultant ground reaction force vector) [16]. Hence, postural regulation constantly tends to counteract perturbations moving the CoM from its initial position. These stabilisation mechanisms are achieved through modulation of muscle stiffness and active intervention from the central nervous system [17], [18]. Consequently, one could expect these mechanisms to oppose a potential effect which would tend to increase body sway when observing human postural imbalance.

In this study we addressed the question of motor contagion when viewing postural imbalance. We recorded participants' body sway while they observed a fixation cross (Control condition), an Upright point-light display of a gymnast balancing on a rope (Upright condition), and the same point-light display presented upside down (Inverted condition). The Inverted condition was designed to distinguish between postural contagion and optical flow effects because of the well described inversion-related impairment in biological motion perception [19], [20]. This enabled us to verify that any recorded postural perturbation in the Upright condition were not merely due to object displacement in the visual field. As a possible postural response, three different hypotheses could be proposed: (1) no influence of the visual stimuli leading to similar body sway in all conditions; (2) an influence of both the Upright and Inverted stimuli due to optical flow and leading to increased (or decreased) body sway compared to the Control condition; (3) an influence of the Upright stimulus only, indicating that only this display carried a biological meaning. Supposedly this could lead to either an increase in body sway due to contagion effects, or a decrease in body sway due to inhibitory and postural regulation mechanisms.

## Methods

### Ethics statement

The experimental protocol was carried out in agreement with legal requirements and international norms (Declaration of Helsinki, 1964) and was approved by the Comité d'éthique régional de Bourgogne which is one ethical reference of Dijon's hospital and of l'Université de Bourgogne. The participants received explanation about the experimental procedure but not about the hypothesis of the experiment. Importantly the displays were not described to them. Written, informed consent was obtained from all subjects.

### Participants

Twenty young subjects (9 females, 11 males; mean age  = 24.5±5.0 years; range  = 21–43) from the Université de Bourgogne (Dijon, France) participated in the study. All had normal or corrected-to-normal vision and none had postural disorders.

### Stimuli

#### Construction of the stimuli

**Figure 1:**
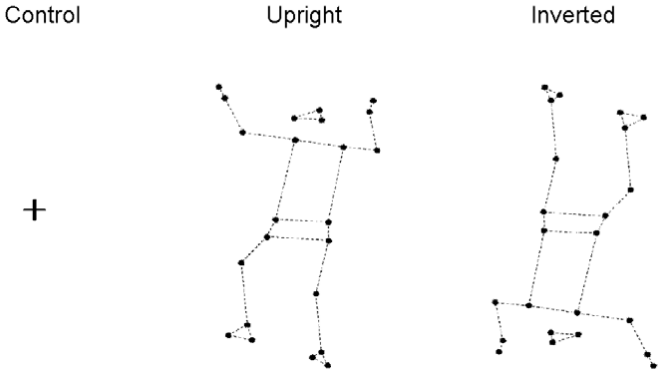
The three stimulus conditions. The Control stimulus was a little white cross presented against a black background. The Upright stimulus was a point-light display of a gymnast, oriented upright, trying to keep his balance on a rope (which was not represented). The biological humanoid was constituted of 23 azure dots (3 for the head, 2 for the pelvis, 1 for each hand, 2 for each foot and 12 for the main joints) presented against a black background. The Inverted stimulus was the same as the Upright one, but rotated 180° around the barycentre of the dots. All stimuli lasted 11s. All displays were shown in reverse contrast and the underlying skeleton, indicated here by the dashed lines, was not represented.

In contrast with previous research on spontaneous imitation, observers were presented with a computer generated display carrying poor visual context, less likely to induce socially motivated imitation. This impoverished display was designed in order to examine specifically the effect of movement kinematics on the contagion process. The stimuli were obtained from the recordings of a gymnast performing a highly unstable postural task. Precisely, the model's task consisted of keeping his balance on a metallic cord (his feet being positioned perpendicularly to the rope). He was equipped with 23 reflexive markers placed on the main joints. The markers were placed on the head (one on each temple and one on the occipital bone, at equal distance from the two other markers), the upper limbs (acromial process of the shoulder, lateral condyle of the elbow, styloid process of the wrist, hand third metacarpophalangeal), the pelvis (anterior superior iliac spine), and the lower limbs (greater trochanter, knee interstitial joint space, ankle external malleolus, calcaneus, foot fifth metatarsophalangeal). The model's movements were recorded with an optoelectronic motion capture system (ELITE, Bioengineering Technology & Systems, Milan, Italy) including eight infrared emitting cameras (sampling rate  = 100 Hz). After acquisition, markers' displacements were smoothed with a Hanning filter in order to eliminate high-frequency noise. From the resulting dynamic point-light (23 points) motion displays, a sequence of 11 seconds was selected, which constituted the Upright biological stimulus (the rope was not materialized). The Inverted biological stimulus was then created by rotating 180° the overall array of point-lights around the barycentre of the dots in a fronto-parallel plane (see Fig. 1). We used the Inverted stimulus in order to distinguish the impact of optical flow from motor contagion. In effect, optical flow was shown to enhance body sway [21]; the Inverted stimulus enabled us to test this effect with a display that is equivalent to the Upright one, but for which we inverted the timing of velocity profiles and destroyed the biological meaning [22]. Precisely, we expected inversion-related impairment in point-light motion recognition [19], [20] to prevent contagion effects. Moreover we could test specifically the effect of integrating gravity constraints in body kinematics by comparing the impact of Upright and Inverted displays on observers' body sway.

#### Projection of the stimuli

The biological stimuli were rear-projected on a translucent 2 m×2 m screen, by a CRT video projector. The spatial resolution of the visual display system was 1024×768 pixels with a vertical refresh rate of 60 Hz. We developed a specific software in C++ to generate the stimuli. The 23 dots constituting the biological stimuli had a diameter of 1 cm each and were displayed in blue against a black background. The point-light model was shown in a 45° rear view. At its fullest extension, it subtended a visual angle of 58° vertically (max distance between a hand and a foot ≈200 cm) and 31° horizontally (max distance between the right and left hand ≈100 cm) at the viewing distance of the subjects (180 cm). The Control stimulus was a little white cross (2 cm width) presented against a black background at a height of 160 cm from the floor.

### Design and Procedure

During the experiment, subjects stood barefoot on a force platform (Techno Concept, France) placed at a distance of 160 cm from the screen, with their feet axes forming an angle of 30° (distance between the heels  = 2 cm) and with their arms alongside their body. The platform recorded excursions of the Centre of Pressure (CoP). Stimuli projection and CoP measurements were synchronized by means of two computers linked by a network. Subjects were instructed that they would see different visual animations and that they had to observe them carefully in order to answer some questions at the end of the experiment. We also emphasized on the fact that they should try to be as relaxed as possible and to focus on the display instead of their own body.

The recordings always began with the display of the white cross (with ambient lights turned on) which served as the Control stimulus. Two separate 11 seconds recordings of CoP displacements were made at this time. Then, with lights off and after a few seconds to let the subjects habituate to darkness, the biological stimuli were presented, each one lasting 11 s and being separated from the other by a 10 s period during which subjects gazed at the white cross. Nine subjects viewed the Upright stimulus first (group Up-first) and nine viewed the Inverted stimulus first (group Inv-first). The duration of the displays was chosen to be short (11 s) compared to the general duration of posturographic studies [23] because we aimed to focus on immediate reactions.

The experiment was performed in a small soundproofed room, temperature regulated (22±1.5°C) and free from external distractions, in which only the investigator and the participant were present. After the session, subjects were asked for each biological stimulus, 1/if the point-light model was human or not 2/what the model was doing, and 3/if they had felt any perturbation of their own posture during observation.

### Data Processing

#### Data analysis

Displacements of the CoP were recorded for the entire duration of each stimulus. However, we removed the first 2 s of each recording from analysis to avoid any potential effect of the abrupt stimulus appearance on subjects' postural sway. For the Control stimulus (white cross), data of the 2 initial recordings were averaged to obtain baseline values (see *Design and Procedure*).

Signals from the force platform were sampled at 40 Hz, amplified, converted from analogue to digital form and stored for off-line analysis using PostureWin software (Techno Concept, France). Prior to analysis, CoP data was filtered with a second-order low-pass Butterworth filter (cut-off frequency: 10 Hz). For each subject and stimulus condition, two postural parameters were then calculated: i) the length of CoP excursions along the 2 axes in the horizontal plane (medio-lateral: M-L; antero-posterior: A–P); ii) the area encompassed by these displacements (computed as the surface of the confidence ellipse containing 90% of the CoP sampled positions).

Furthermore, because there is a strong coupling between the displacements of CoP and Centre of Mass (CoM) during Upright posture [17], [24], we looked for potential links between subjects' CoP excursions and the displacements of the model's CoM. As mentioned before, the biological stimuli were constructed with markers placed on the whole body of a subject performing the equilibrium task. By means of these landmarks and anthropometric data, a 9-segment model (2 legs, 2 thighs, 1 pelvis-trunk, 2 arms and 2 upper arms) was developed to estimate the total body CoM. The model was inspired from a previous study [25]; however the total number of markers differed.

The CoM was the weighted average of each of the CoM of the 9 segments:
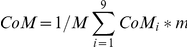



Where M is the total body mass, m_i_ is the mass of the _i_th segment, and CoM_i_ is the centre of mass of the _i_th segment.

The degree of coupling between the displacements of the subjects' CoP and the model's CoM was assessed in both the A-P and M-L axes of the horizontal plane by means of cross-correlation analysis. This analysis was performed for each subject and stimulus condition and the absolute value of the peak correlation coefficient was taken into account (using a time lag of ±2 s).

#### Statistical analysis

Statistical analyses were computed using Statistica 6 (Statsoft, Tulsa, OK). A repeated measures ANOVA was conducted on each parameter with Group (Up-first, Inv-first) as between-subjects factor, and Stimulus (Control, Upright and Inverted) as within-subjects factor. Scheffé tests were used for post hoc comparisons and p-values <0.05 were considered significant for all statistical analyses.

A complementary analysis was conducted on each parameter with Group (Up-first, Inv-first) as between-subjects factor, and Stimulus (Control and Upright for group Up-first/Control and Inverted for group Inv-first) as within-subjects factor. These analyses were performed in order to assess the effect of each display that would not be influenced by a previous stimulus. Scheffé tests were again used for post hoc comparisons and p-values <0.05 were considered significant.

## Results

The data of two subjects (2 females) were not included in the analysis because they reported having tried to stand as straight as possible during the stimuli in spite of being explicitly instructed not to try to voluntarily control their body. Thus, statistical analyses were carried out on the mean data of 18 subjects.

### Subjects' body sway

**Figure 2:**
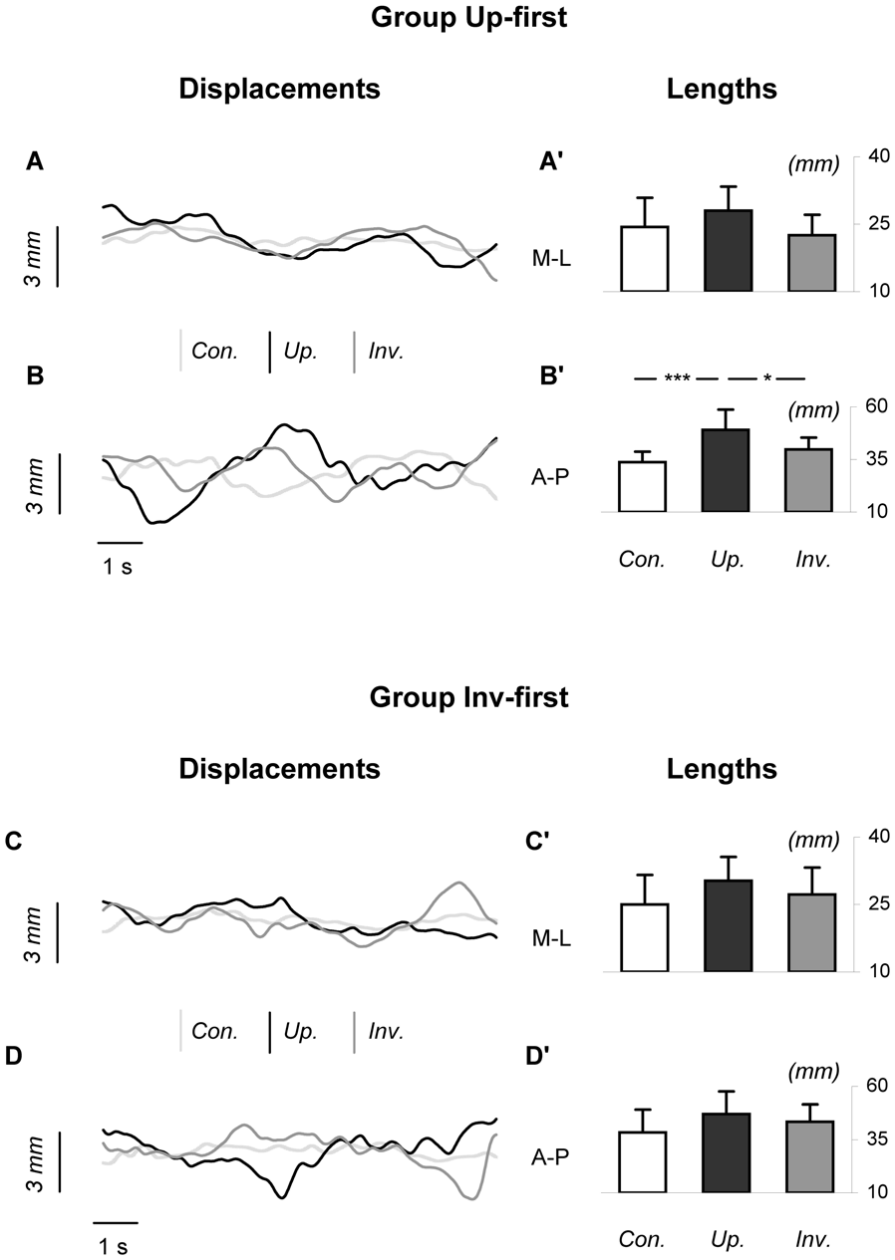
Representative excursions and mean lengths of CoP displacement in the two axes. Panels A and B show CoP excursions of a representative subject from group Up-first in the three stimulus conditions in the medio-lateral (M-L) and antero-posterior (A-P) directions. Panels A' and B' represent mean lengths of CoP displacement in both axes for group Up-first. Panels C, D, C', D' represent the corresponding data for group Inv-first. Bars indicate standard deviations; * and *** mean significant differences between conditions (respectively p<0.05 and p<0.001; ANOVA for repeated measures). Con.  =  Control condition, Up.  =  Upright condition, Inv.  =  Inverted condition. As can be seen for group Up-first, the length of A-P CoP excursions was greater in the Upright condition compared to the two other conditions.

For each group, figure 2 represents CoP excursions of a typical subject, as well as the mean displacements of subjects' CoP along the two axes (M-L, A-P) of the horizontal plane, during the three experimental conditions.

The ANOVA performed on A-P CoP displacement showed a significance of the factor Stimulus only (F(2,32)  = 21.43; p<0.001). More specifically, post-hoc analysis indicated that: i) for group Up-first, A-P displacement was greater for the Upright condition (48.98±9.63 mm) compared to the two other conditions (Control: 33.68±4.99 mm, p<0.001; Inverted: 39.72±5.61 mm, p<0.05) whereas no statistical difference was present between the Control and Inverted conditions (p = 0.39); ii) for Group Inv-first, no significant difference was detected between the Upright (47.00±10.57 mm), Inverted (43.42±8.16 mm) and Control conditions (38.35±10.76 mm).

The ANOVA performed on M-L CoP displacement also showed a significance of the factor Stimulus (F(2,32) = 7.17; p<0.01). However post-hoc analysis revealed no significant difference between conditions.

**Figure 3:**
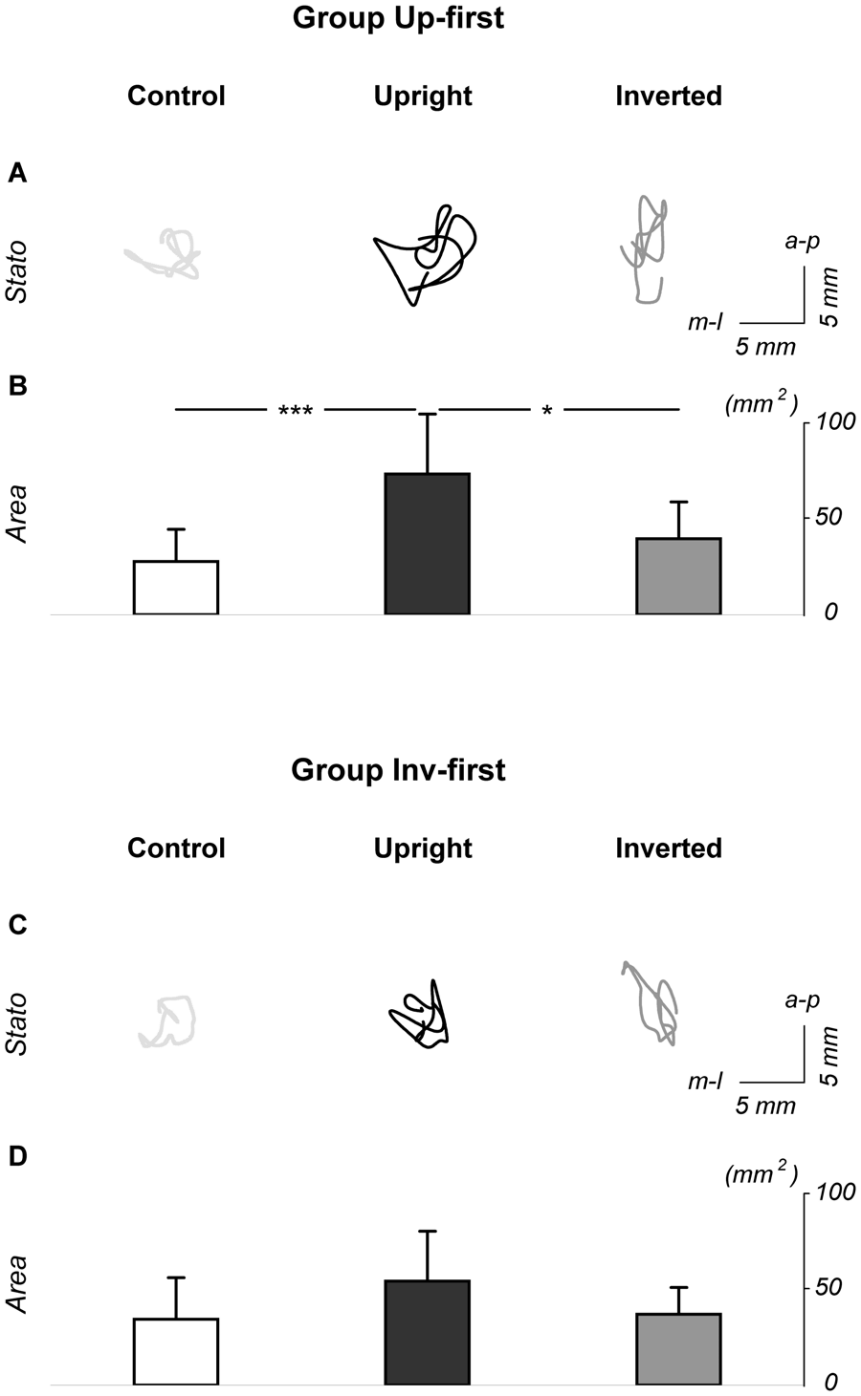
Area of CoP excursions in the three stimulus conditions. Panel A shows statokinesigrams of the CoP for a typical subject from group Up-first. Panel B shows the mean area of CoP displacement (area of the confidence ellipse including 90% of the distribution of the CoP) in the three stimulus conditions for group Up-first. Panels C and D illustrate the corresponding data for group Inv-first. Bars indicate standard deviations, * and *** mean significant differences between conditions (respectively p<0.05 and p<0.001; ANOVA for repeated measures). As can be seen for group Up-first, the mean area of CoP displacement was greater in the Upright condition compared to the two other conditions.

For each group, figure 3 shows representative statokinesigrams of a typical subject, and average values of areas encompassed by subjects' CoP excursions during the three stimulus conditions. The ANOVA revealed a significance of the factor Stimulus (F(2,32) = 18.31; p<0.001) and a tendency to an interaction Group x Stimulus (F(2,32) = 2.66; p = 0.09). More specifically, post-hoc analysis indicated that: i) for group Up-first, area covered by CoP excursions was greater for the Upright condition (70.31±29.96 mm^2^) compared to the two other conditions (Control: 26.54±16.11 mm^2^, p<0.001; Inverted: 37.98±18.29 mm^2^, p<0.05), whereas no statistical difference was present between the Control and Inverted conditions (p = 0.82); ii) for Group Inv-first, no significant difference was detected between the Upright (51.98±24.93 mm^2^), Inverted (35.48±13.34 mm^2^) and Control conditions (33.01±20.70 mm^2^).

**Figure 4:**
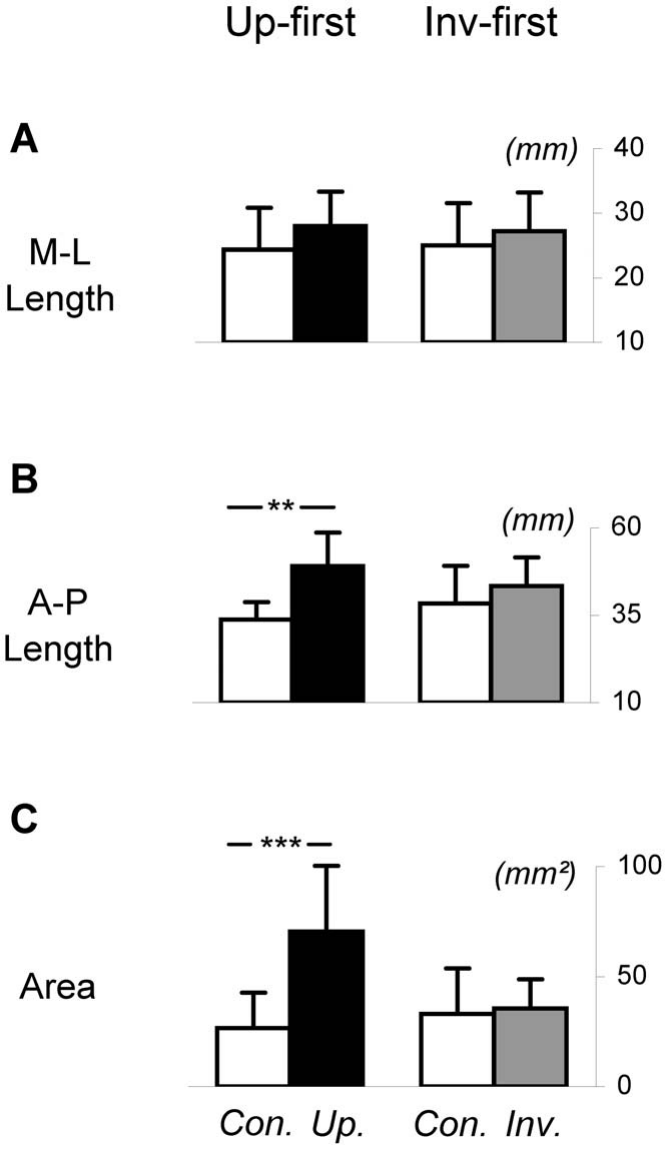
Mean lengths and area of CoP excursions: comparison between groups Up-first and Inv-first. Panels A, B and C respectively show mean medio-lateral length, antero-posterior length and area covered by CoP excursions in the Control and Upright conditions for Group Up-first, and Control and Inverted conditions for group Inv-first. Bars indicate standard deviations, ** and *** mean significant differences between conditions (respectively p<0.01 and p<0.001). Con.  =  Control condition, Up.  =  Upright condition, Inv.  =  Inverted condition. As can be seen, A-P CoP displacement and CoP area were significantly higher in the Upright condition compared to the Control condition for Group Up-first. No significant difference was detected between the Inverted and Control conditions for group Inv-first.

Figure 4 compares groups Up-first and Inv-first considering only the Control condition and the first stimulus displayed for each group, thus avoiding potential after-effects of the displays. The ANOVAs conducted here confirmed previous results. More precisely, the analysis performed on A-P CoP displacements showed a significance of the factor Stimulus (F(1,16) = 22.58; p<0.001) and a significant interaction Group x Stimulus (F(1,16) = 5.85; p<0.05). Further, post-hoc analysis revealed that: i) for group Up-first, A-P displacement was greater for the Upright compared to the Control condition (p<0.001); ii) for group Inv-first, there was no statistical difference between the Inverted and Control conditions (p = 0.46).

The analysis performed on M-L CoP displacements revealed no significant effect.

Finally the ANOVA performed on CoP area showed a significance of the factor Stimulus (F(1,16) = 21.30; p<0.001) and a significant interaction Group x Stimulus (F(1,16) = 16.99; p<0.001). Post-hoc analysis revealed that: i) for group Up-first, A-P displacement was greater for the Upright compared to the Control condition (p<0.001); ii) for group Inv-first, there was no statistical difference between the Inverted and Control conditions (p = 0.99).

To sum up, we found an increase of subjects' body sway when they observed the Upright stimulus as compared with Inverted and Control ones. Although this increase is graphically apparent for both groups, it was significant only for group Up-first.

Cross correlation analyses allowed the comparison of the excursions of subjects' CoP with the displacements of the model's CoM, during the different conditions. The ANOVAs performed on the mean peak correlation coefficients did not reveal any significant difference between conditions, either in the A-P or M-L axis. Thus, subjects' CoP excursions were not particularly correlated with the Model's CoM displacements for the three experimental conditions.

### Impact of ambient light

In order to assess the impact of ambient light in the Control condition (see *Methods*, *Design and Procedure*), a control experiment was conducted in which we presented 9 subjects (3 females, 6 males; age: 25.9±7.25 years; range: 21–43) with a fixation cross in a light ambient (2×11 s) followed by a fixation cross in a dark ambient (2×11 s). Mean area, medio-lateral and antero-posterior CoP path lengths were calculated for each subject and condition, considering the last 9 seconds of each recording trial, as in the main experiment (see *Methods*). Subjects' responses to the fixation cross in light vs. dark ambient were then compared by means of paired t-tests. No significant difference was detected between conditions ([Area: cross/light: 28.34±23.55 mm^2^; cross/dark: 29.30±11.93 mm^2^; p = 0.90]; [M-L displacement: cross/light: 27.11±11.08 mm; cross/dark: 23.98±5.91 mm; p = 0.16]; [A-P displacement: cross/light: 32.63±11.33 mm; cross/dark: 39.22±11.09 mm; p = 0.07]). These results indicate that light was not a predominant factor to affect body sway in these experimental conditions.

### Subjects' reports

All subjects recognized without hesitation the Upright stimulus as a human model. Interestingly, and contrarily to what we expected [26], [27], 15 out of 18 subjects also identified the Inverted stimulus as human. However, when asked to describe the action performed by the model, some differences appeared between the Upright and Inverted conditions. For the Upright presentation, even if the interpretation of the movement performed by the model could vary across subjects (some of them recognized a man on a rope, some others a dancer), all participants clearly mentioned that the model was in postural disequilibrium. For the Inverted condition, i) subjects who had seen the Upright presentation before (group Up-first, 9 out of 18 subjects) identified the Inverted stimulus as being the same as the Upright one; ii) subjects who had seen the Inverted stimulus first did not identify the action performed by the model (group Inv-first, 9 out of 18 subjects).

Finally, when asked about their own body sway, 7 subjects (4 from group Up-first; 3 from group Inv-first) declared having felt little changes of their posture associated with the observation of the biological stimuli (both in the Upright and Inverted positions). The remaining 11 subjects (5 from group Up-first; 6 from group Inv-first) did not feel anything particular. These reports indicate that the subjects were not aware of the different postural effects induced by the Upright and Inverted displays.

## Discussion

This study demonstrates that observing an Upright point-light display of postural imbalance induces an increase of observers' body sway. We detected larger areas of CoP excursion and larger A-P CoP displacements in the Upright condition when compared to Inverted and Control ones. These results were significant only for subjects who viewed the Upright stimulus prior to the Inverted one (group Up-first). In general, our results extend previous work that showed body limb re-orientation after displaying postural imbalance of a meaningful human model [28] or an avatar [29]. However, different body limb configurations can be associated to the same CoM position, and do not reflect postural control which is only visible through CoP and CoM displacements. For instance, both CoM stabilisation and destabilisation can result from large upper limb adjustments.

Otherwise, the correlation analysis between observers' CoP displacement and the model's CoM displacement did not reveal any temporal link between stimulus and subjects' motion. This suggests that observers' postural sway did not match the model's imbalance in a one-to-one mapping. Nevertheless, the Upright stimulus predominantly affected observers' posture along the antero-posterior axis, that is the axis along which the model mainly oscillated.

Importantly, the point-light stimuli used in this study represented an impoverished visual context without materialization of the support (rope), which may have penalised recognition and goal identification. However, all observers identified the Upright stimulus as a human model in postural imbalance indicating that kinematic information, rather than the visual context, is tuning motion recognition [22]. Accordingly, a study demonstrates that biological motion recognition is mainly based on local kinematics of body parts displacement, besides the global processing that reconstructs a coherent body structure [30]. For instance, during locomotion, foot trajectory (as the principal body part interacting with gravity and the base of support) is the most relevant information for a direction discrimination task [20].

When reversing the display (Inverted condition), CoP excursions were not significantly different from the Control condition, suggesting that the optical flow was not sufficient to enhance body oscillations. Further, this indicates that the postural effect of the Upright display was gravity dependent. In the Inverted condition, body kinematics was violating the gravitational force field, and the stimulus was more similar to a stable pendulum instead of the unstable inverted one characterizing bipedal human posture [17], [31]. It is well known that action planning and execution are strongly dependent on the gravitational force field. Indeed, arm and body motion velocity profiles in the sagittal plane are asymmetric, in contrast to horizontal movement [32], [33], [34]. Consequently, when inverted, these velocity profiles produce nonbiological kinematics.

Noticeably, based on subjects' reports, we found that Inverted display recognition was modulated by the previous display of the Upright model. Precisely, when the Inverted model followed the Upright one (group Up-first), subjects better recognized both, the human configuration and the equilibrium task. In contrast, in the opposite order of appearance (group Inv-first), subjects recognized only the body configuration (an “inverted human picture”), as classically shown for inverted locomotion display [35].

Interestingly, postural contamination was also found dependant on presentation order. The postural effect recorded in group Up-first suggests that contagion mechanisms were promoted by action recognition and observer/model interaction in the Upright condition. Still in group Up-first, despite recognition of the Inverted model, no significant effect of the Inverted display was detected, suggesting that: 1) optical flow presented in this display was insufficient to affect observers' posture, 2) motor contamination of the Inverted display was prevented by non biological kinematics, and 3) the previous Upright display may have induced a stabilisation response that limited observers' reaction to the Inverted stimulus. As concerns group Inv-first, no significant postural effect was detected for either the Upright or Inverted display, which would imply that visual information first presented in the Inverted display, although insufficient to contaminate observers' posture, provoked a stabilisation reaction which prevented contagion by the subsequent Upright stimulus. This restriction of postural effects was efficient despite recognition of the human shape and action performed by the Upright model.

Overall the motor/postural effects found here are in contrast with the well documented absence (or subliminal presence) of overt motor activation during movement observation [13]. The present result suggests a partial inefficiency of inhibitory processes [12], as it was parsimoniously observed during explicit motor imagery [38]. Nevertheless observers were able to keep a stable standing position, indicating that stabilisation mechanisms which are mostly controlled at a subcortical level in the brain stem and cerebellum [39], were able to prevent a significant loss of equilibrium. In line with our results, vegetative output such as respiration rate, devoid of voluntary control, was modulated during observation of an effortful action [13,40,41]. In the same vein, the display of pictures of negative emotional valence was shown to induce a postural effect (in this case a freezing reaction) [42,43].

Human standing posture, that is automatic and mainly subcortically controlled, is somehow comparable to homeostatic regulation [39]. Thus, inhibitory processes could be less efficient to prevent contagious outputs on low-level functions. An alternative interpretation, still compatible with the previous one, is that when the visual stimulus contains some emotionally-relevant connotation (here a possible fall), mechanisms preventing overt imitation of observed actions could be partly de-inhibited. At last, it has been suggested that contagion effect would be mediated by the mirror neuron system (MNS) in the premotor or parietal regions [36]. Precisely, the human MNS responds to both executed and observed actions [37], and therefore might mediate the interference between executed and observed actions. In the present case, body oscillations generated by the interference/contagion effect would produce a prediction error via forward connections, that in turn would transiently render inefficient sensory feedback (as predicted by the motor command) to postural stabilization.

### Conclusion

This study demonstrates that observing the point-light display of a model in postural imbalance increases observers' body sway, indicating that this visual stimulus triggers a postural contagion. This result confirms Lipps' theory, intuitively proposed a century ago, that observers watching an unstable tightrope walker tend to spontaneously imitate this model [44]. Further experiments are however requested to better investigate this issue. For instance, it will be interesting to manipulate viewpoint and direction of imbalance to more clearly identify the mechanisms involved in this postural effect. Enriched virtual environments might as well increase motor outputs and could be used to better contextualize the observed behaviour. From a clinical point of view, the potential contribution of this study is twofold. First, relative to the question of social interactions, this paradigm could possibly bring an additional tool to better diagnose patients with intersubjectivity deficiency, such as autistic syndrome; these subjects would indeed be less likely contaminated by a biological display. On the other hand, this protocol could serve to evaluate postural instability in patients with altered equilibration function. For instance elderly people with increased postural frailty would present more difficulty to compensate for postural contamination. Furthermore, repeated observation of postural imbalance could be introduced as an observational training protocol to improve equilibration strategies. Such practice would require being adapted to each patient in terms of type of stimuli, duration and frequency of exposure, and could be tested as a complementary tool for postural rehabilitation and fall prevention.
