# Correction: Does Observation of Postural Imbalance Induce a Postural Reaction?

**DOI:** 10.1371/annotation/aff2c1f3-08af-4a36-bf53-0df2bfd9c320

**Published:** 2011-03-24

**Authors:** Banty Tia, Arnaud Saimpont, Christos Paizis, France Mourey, Luciano Fadiga, Thierry Pozzo

The authors would like to add the following sentence to the Funding section: "L. Fadiga was supported by the EU project SIEMPRE."

